# Application of electronic nose and machine learning used to detect soybean gases under water stress and variability throughout the daytime

**DOI:** 10.3389/fpls.2024.1323296

**Published:** 2024-04-05

**Authors:** Paulo Sergio De Paula Herrmann, Matheus dos Santos Luccas, Ednaldo José Ferreira, André Torre Neto

**Affiliations:** ^1^ Embrapa Instrumentation, São Carlos, Brazil; ^2^ Institute of Mathematical and Computer Sciences, University of São Paulo, São Carlos, Brazil

**Keywords:** E-nose, water stress, non-invasive phenotyping, artificial intelligence, data mining, soybean

## Abstract

The development of non-invasive methods and accessible tools for application to plant phenotyping is considered a breakthrough. This work presents the preliminary results using an electronic nose (E-Nose) and machine learning (ML) as affordable tools. An E-Nose is an electronic system used for smell global analysis, which emulates the human nose structure. The soybean (Glycine Max) was used to conduct this experiment under water stress. Commercial E-Nose was used, and a chamber was designed and built to conduct the measurement of the gas sample from the soybean. This experiment was conducted for 22 days, observing the stages of plant growth during this period. This chamber is embedded with relative humidity [RH (%)], temperature (°C), and CO_2_ concentration (ppm) sensors, as well as the natural light intensity, which was monitored. These systems allowed intermittent monitoring of each parameter to create a database. The soil used was the red-yellow dystrophic type and was covered to avoid evapotranspiration effects. The measurement with the electronic nose was done daily, during the morning and afternoon, and in two phenological situations of the plant (with the healthful soy irrigated with deionized water and underwater stress) until the growth V5 stage to obtain the plant gases emissions. Data mining techniques were used, through the software “Weka™” and the decision tree strategy. From the evaluation of the sensors database, a dynamic variation of plant respiration pattern was observed, with the two distinct behaviors observed in the morning (~9:30 am) and afternoon (3:30 pm). With the initial results obtained with the E-Nose signals and ML, it was possible to distinguish the two situations, i.e., the irrigated plant standard and underwater stress, the influence of the two periods of daylight, and influence of temporal variability of the weather. As a result of this investigation, a classifier was developed that, through a non-invasive analysis of gas samples, can accurately determine the absence of water in soybean plants with a rate of 94.4% accuracy. Future investigations should be carried out under controlled conditions that enable early detection of the stress level.

## 1 Introduction

Abiotic stress is a term used to describe a range of environmental stresses that can affect crops, such as elevated temperature, chilling, excessive light, drought, waterlogging, wounding, exposure to ozone, UV-B irradiation, osmotic shock, and salinity. According to Bray et al. (2000), abiotic stress can lead to a potential yield loss of 51-82% in annual crops.

Zhao and collaborators in their investigation predict that significant crop yields, such as wheat, rice, corn, and soybeans, will decrease by an average of 6.0%, 3.2%, 7.4%, and 3.1%, respectively, for each degree Celsius increase in the global average temperature (Zhao et al., 2017).

The present moment demands careful consideration to improve the knowledge about biotic and abiotic stress to sustainable agriculture, food security, population growth, and the efficient use of natural resources, necessitating multidisciplinary and interdisciplinary research. As a result, collaboration among various fields, such as engineering, physics, geosciences, plant sciences, ecophysiology, computer science, and instrumentation, is crucial to developing effective non-invasive plant phenotyping techniques and methods. In agriculture, the key to practical applications lies in affordable, lightweight, and adaptable devices, instruments, sensors, and biosensors. The current trend in phenotyping research favors non-invasive techniques (Fiorani and Schurr, 2013).

Land vegetation accounts for 90% of global VOC emissions (Kiendler-Scharr et al., 2009). Plants emit volatile organic compounds (VOCs) when they suffer from disease, making them an ideal measure of phenotypic dynamics with promising results (Niederbacher et al., 2015).

**Figure 1:**
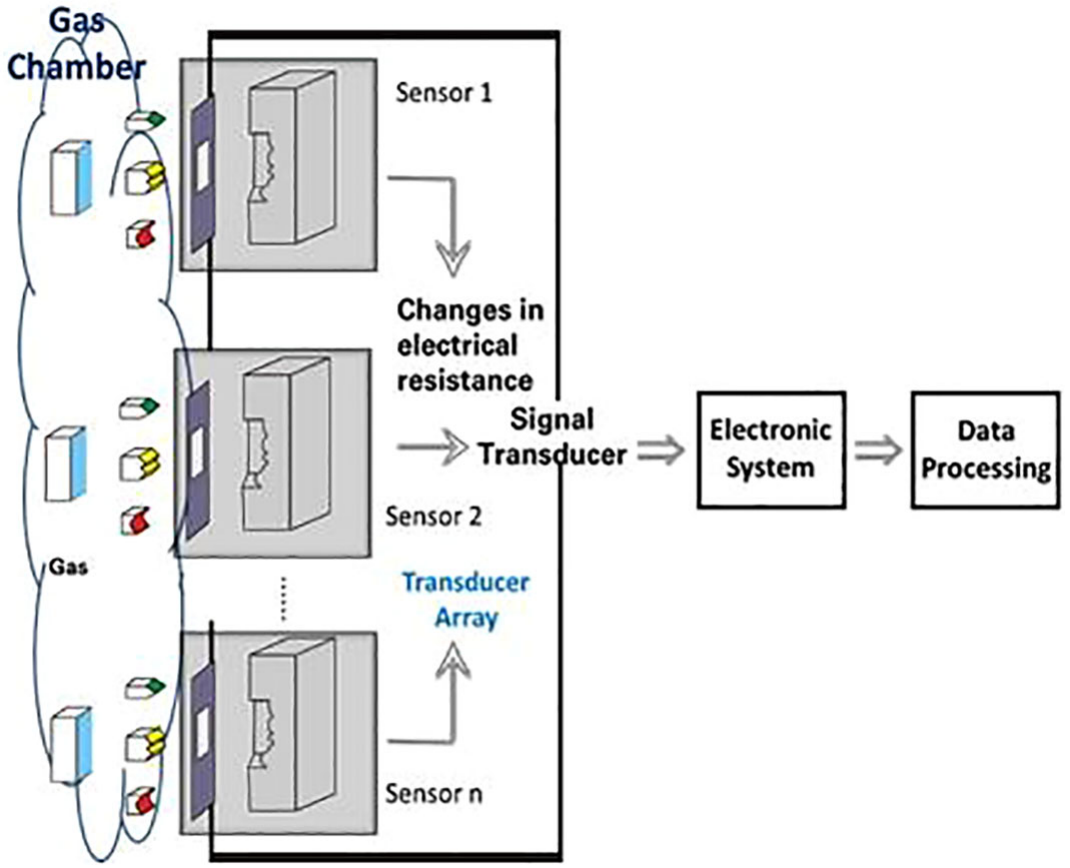
The block diagram of an E-nose and its components, including sensors, signal transducer, electronic system, and data processing.

Affordable plant gas detection methods could soon include electronic nose (E-Nose) and A.I. applications. The concept of electronic nose was first introduced in 1982 by Persaud and Dodd at the University of Warwick (Persaud and Dodd, 1982). Gardner and Bartlett (1994) provided the most accepted definition, defining the system as “an instrument comprised of an array of electronic sensors with specific recognition capabilities and a standard recognition system that can detect olfactory substances ranging from simple to complex” (Schaller et al., 1998). The olfactory system is more complex than other sensory systems like vision and hearing, with hundreds of different biological sensors involved in olfaction. Each olfactory receptor cell has only one type of odor receptor, which can detect only a limited number of substances (Lozano et al., 2005). **Figure 1** illustrates a block diagram of the E-Nose concept. E-noses have been widely used and studied by large companies in industries such as food, cosmetics, packaging, pharmaceuticals, chemicals, petrochemicals, and agriculture (Manzoli et al., 2011; Steffens et al., 2014). This technology is a fast, simple, low-cost, and non-destructive tool for quality control and decision-making. In medicine, it has been used to detect chemicals in lung cancer patients and monitor the fertile period of cows in livestock (de Vries et al., 2019). In agriculture, monitoring insects and pests with current techniques is time-consuming and often yields variable results, making it challenging for producers and consultants to make reliable and accessible decisions. Electronic noses can use distinct types of sensors, including conductive polymers, “Carbon Black,” and carbon nanotubes (Manzoli et al., 2019; Garcia-Berrios et al., 2013; Chatterjee et al., 2013).

Hazarika and collaborators presented in their investigation showed a technique to detect a pathogen called *Citrus Tristeza Virus* (CTV) in Khasi mandarin plants, where the biological process of smell was mimicked by electronic nose (E-Nose). They used invasive and destructive methods, where leaf samples were cut with scissors into square pieces measuring approximately 1 cm by 1 cm and placed in the sample holder. To evaluate the signal from the E-Nose was used classifier models such as bagging k-nearest neighbors (KNN Bag), adaptive boosting (AdaBoost) decision tree (ABDT) (Hazarika et al., 2020) and deep neural network (DNN) (Sharma et al., 2023).

Water stress triggers various physiological and biochemical responses in plants, such as stomatal closure, growth and photosynthesis repression, and respiration activation (Hale and Orcutt, 1987).

Environmental factors that affect transpiration change the water vapor gradient between the leaf surface and surrounding air: the energy balance between the sun and the leaf, air humidity and temperature, wind, and soil water availability (Angelocci et al., 2004). Therefore, transpiration intensifies with decreasing relative humidity and increasing air temperature.

Plant transpiration is vital because it goes beyond eliminating excess water and accelerates the transport of raw sap. The sap is a nutrient-rich substance, from the root to the leaves. It is transformed into an elaborate sap related to the plant’s production. Transpiration assessment was used as a direct quantitative relationship of water status in vines, and this parameter was used as an indicator to organize the plant’s irrigation schedule (Patakas et al., 2005).

Transpiration is the evaporation of water from plant leaves. Transpiration involves vaporizing liquid water in plant tissues and removing the vapor into the atmosphere. Crops lose water through stomata.

The relationship between transpiration and water stress can be measured using a variety of methods. One common method is to measure the rate of water loss from a plant leaf using a potometer (Pasqualotto et al., 2019). Another method is to measure the leaf water potential, which is a measure of the amount of water that is available to the plant (Ratzmann et al., 2019), and others, as for example the lysimeter. They all have limitations, disadvantages, high-cost and can sometimes produce incorrect results. However, the potential of electronic nose applications can overcome these difficulties and present a new exploration technique and method.

Sinclair et al. (2010) showed in their work the relations of vapor pressure deficit (VPD) and how it affected the sensitivity of the transpiration (TR): the time hours of Low VPD are between 7 – 11:00 a.m. and High VPD are between 11:00 a.m. – 3:00 p.m.

Studies have shown that water stress can significantly reduce the rate of transpiration in soybean plants, which can impact the plants’ overall health and productivity (Lambers et al., 2008).

An increase in atmospheric CO_2_, in terms of transpiration, or water use by the plant, means that the stomata, or the leaf pores that exchange gases between the leaf and the atmosphere, do not need to open as much. The effects can occur at the level of abiotic and biotic stress. Work by Sun and collaborators showed that with an increase in CO_2_, the leaf transpiration rate (mmol H_2_O m-2 s-1) decreased and the work showed the influence on the infestation of pea aphid (*Acyrthosiphon pisum*) in Medicago truncatula (Sun et al., 2015).

Soybean crops are highly vulnerable to the detrimental effects of drought, an abiotic stress that can cause severe damage to the plant’s growth and development. This stress is particularly impactful during certain stages of the soybean’s life cycle, leading to substantial yield losses. Studies have shown that soybean’s sensitivity to drought is relatively high, with annual losses of up to 40% attributed to this kind of stress (Basal and Szabó, 2020).

The precise effects of water stress on soybean physiology and biochemistry remain unclear. Insufficient soil moisture triggers a range of plant adaptations, including morphological, physiological, and biochemical processes that can inhibit growth, lower photosynthesis, and transpiration rates, diminish chlorophyll levels, and modify protein structures. Given the complex nature of photosynthesis and gas exchange, these processes serve as valuable indicators of soybean response to soil moisture stress during the vegetative phase (Wijewardana et al., 2019).

The process of measuring gas exchange in leaves often involves interfering with their natural physiology, as it requires direct contact with the leaves.

Machine learning is a combination of data science and statistics that is based on the probability of occurrence of events, patterns, and behaviors in the provided database (Tan et al., 2006; Han et al., 2011). For this project, data mining techniques were employed to enable the machine to study a relevant database and detect stress levels.

Those techniques (E-Nose and Machine Learning) could be a valuable tool for assessing water stress levels in soybeans, serving as a new method of phenotyping plants that can be applied in precision agriculture. It is an affordable device that can be used for global gas analysis. It allows the application of machine learning – the basis of artificial intelligence – to examine data generated due to abiotic plant stress.

Soybeans are of great economic importance to Brazil, the second-largest producer of this crop in the world, and this crop’s success directly impacts the national GDP. In 2018/2019, soybeans occupied an area of 44,062 million hectares, producing 154.566 million tons – resulting in productivity rate of 3,508 kg/ha (Embrapa Soja, 2023).

The electronic nose, through global gas analysis, is being used as a new tool for plant phenotyping, aiming to investigate water stress and the influence of sample acquisition during two separate times of gas acquisition [morning (9:30 a.m.) and (3:30 p.m.)] as well as the use of machine learning to detect the absence of irrigation. The system will provide easy handling, quick response, and a flexible tool for pattern recognition through machine learning and artificial intelligence techniques for severity observations and non-destructive measurements with portability. These differential factors highlight the advantages researchers, producers, and consultants can use in decision-making in favor of crop management.

This project work aims to investigate the use of an electronic nose and machine learning techniques to obtain non-invasive values of transpiration in soybeans (Glycine Max), evaluate the water stress and investigate de temporal variability.

The study conducted experiments proposing innovations in phenotyping, presenting, and enhancing an affordable system that employs E-Nose technology. This system allows for non-invasive and non-destructive studies using automated instrumentation in data collection. It is particularly useful in investigating abiotic effects such as water stress and examining the seasonal influence of gas emissions throughout the day. Additionally, it utilizes data mining and machine learning techniques to extract meaningful information.

## 2 Materials and methods

### 2.1 Electronic Nose Alpha FOX 2000

An Electronic Nose, model Alpha FOX 2000 was used, which also came with several tools in data processing and analysis software, helping in the proposal of joining the device with the Data Mining technology.

The equipment is built with six (06) n-type tin oxide complementary metal oxide semiconductors (CMOS) sensors. The quantity of catalytic metals (platinum palladium) in the tin oxide will be influenced by their selectivity (Vernat-Rossi et al., 1996). **Table 1** lists all sensors and their main applications. They detect the variation of the electrical resistance due to the interaction of the gases with the semiconductor surface (FOX Analyzer - Hardware User’s Guide, 2000).

**Table 1:** The sensors installed in the E-Nose are (Wei et al., 2017).

No.	Sensor	Sensitivity property	Reference Materials
1	T30/1	Organic compounds	Organic compounds
2	P10/1	Combustible gas	hydrocarbon
3	P10/2	Inflammable gas	methane
4	P40/1	Oxidizing gas	fluorine
5	T70/2	Aromatic compounds	methylbenyene, xylene
6	PA/2	Organic compounds and toxic gas	Ammonia, amines, ethyl alcohol

#### 2.1.1 E-nose measurements

The E-Nose FOX 2000 model was configured to acquire data from the variation in electrical resistance (Ω) of every six sensors over time, using the following parameters:

Acquisition duration (s): 240; Acquisition period (s): 1; Acquisition time (s): 300; Flow rate of 150 (ml/min); Injection Volume (µl): 500; Injection speed (µl/s): 500. The internal chamber of the E-Nose, that there are located the sensors of the equipment with an internal temperature adjusted to 64°C and the 0.0 (%) of the relative humidity.

The gas sample, from the chamber, were collected using a Syringe for Headspace 2,500 (µl) H 0,72 (G22) d 51 PTFE seal. The precision:< ± 1% of the volume. The volume used to extract the samples was 500 (µl) to each measurement.

##### 2.1.1.1 Calculation used on the response of the sensors

The sensitivity S (%) for each sensor was calculated using the following Equation (1):


(1) $$S\left(\%\right)=\left(\frac{R-R_{0}}{R_{0}}\right)x100\;\;\;\;\left(\%\right)\;\;\;\;\;\;\;$$

R_0_– Initial electrical resistance (Ω);

R – Electrical resistance varying over time (Ω).

**Figure 2:**
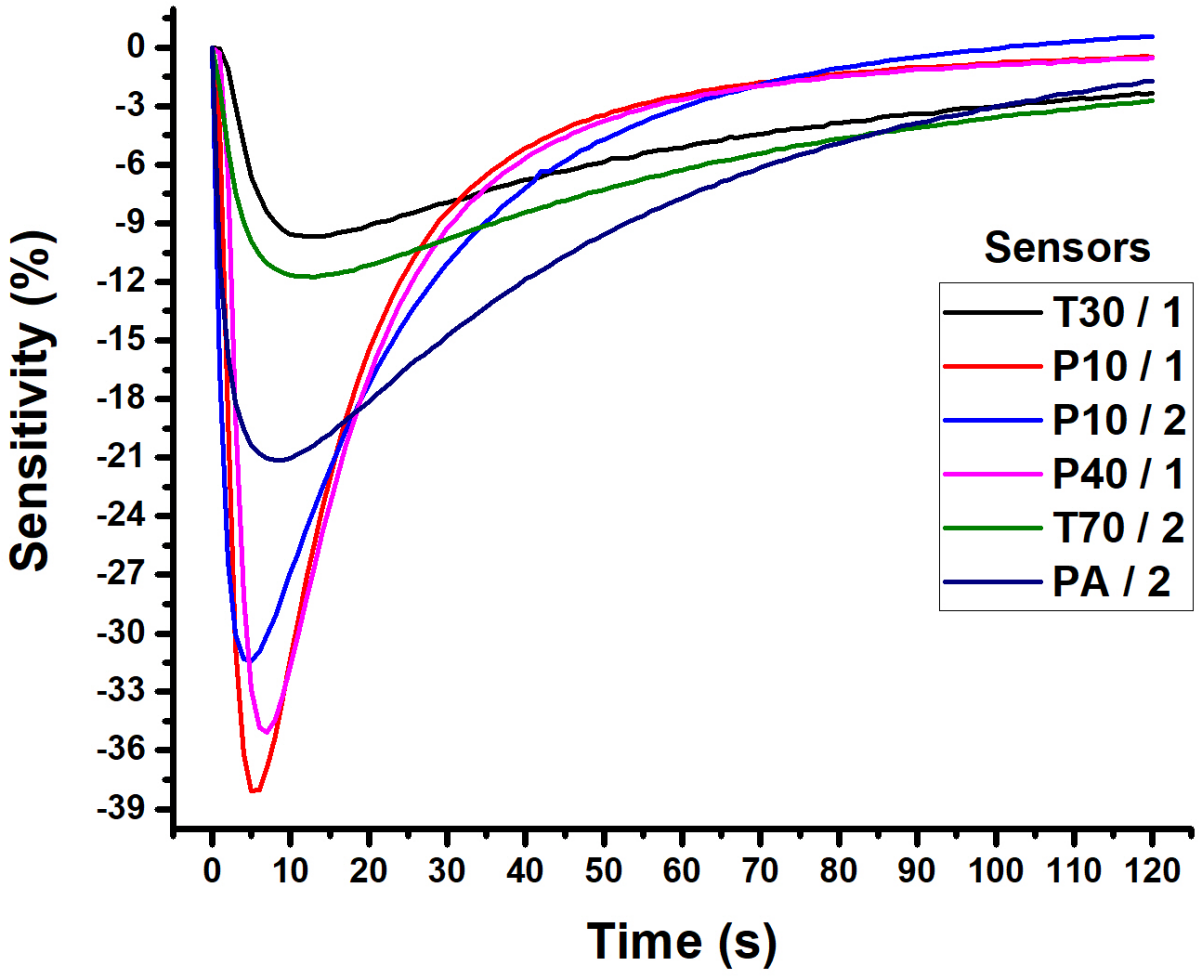
The variation in sensitivity of each of the six sensors in relation to time, depending on the gas sampled and measured in the E-Nose.

To analyze the data that were acquired from the E-Nose, has been used the radar chart and the area radar from the peak of the sensitivity [S (%)] of each six sensors were used (S1: T30/1; S2: P10/1; S3: P10/2; S4: P40/1; S5: T70/2 and S6: PA/2). An area radar chart is a type of radar chart that uses the area enclosed by the lines connecting the data points to represent the values. In **Figure 2**, there is a representation of the radar chart and radar area to the peak of the sensitivity [S (%)]. The negative sign of S (%) shows that the electrical resistance (Ω) of each sensor is decreasing relative to its baseline. The information shows that the sensor is more conductive. A higher numerical value in S (%) indicates that the sensor is more sensitive to the gas sample it is detecting.

**Figure 3:**
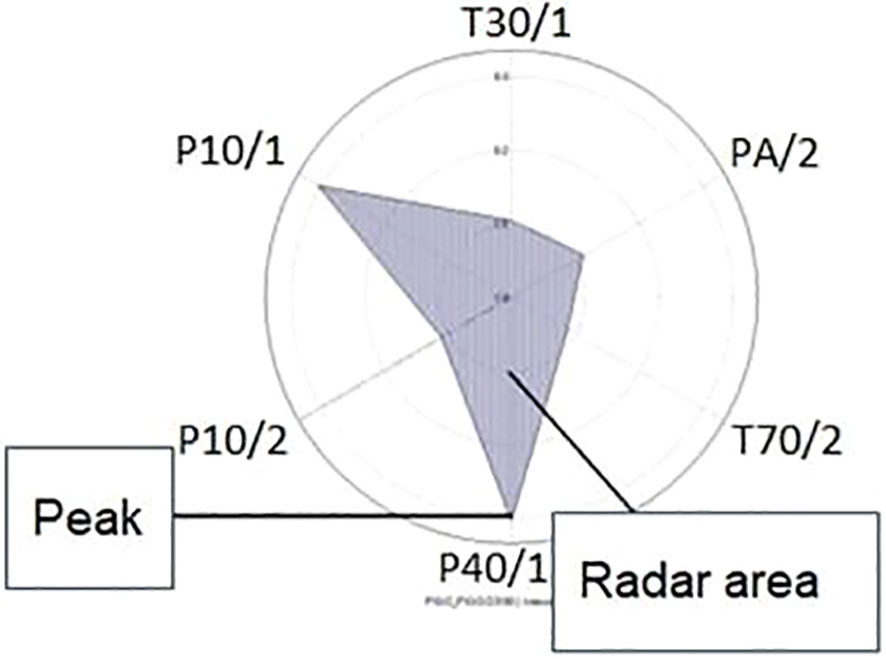
The radar chart and radar area from the sensitivity (%) peak to the six sensors (S1: T30/1; S2: P10/1; S3: P10/2; S4: P40/1; S5: T70/2 and S6: PA/2) from the E-nose.

An area radar chart is a type of radar chart that uses the area enclosed by the lines connecting the data points to represent the values. In **Figure 3**, there is a representation of the radar chart and radar area to the peak of the sensitivity [S (%)]. This can be useful for comparing the overall performance of distinct data groups. Liu and collaborators have used the method that uses radar charts to visualize multi-dimensional data. Radar charts are a type of chart that can be used to represent multiple variables at once (Liu et al., 2008).

A radar chart is a graphical representation that effectively illustrates multidimensional data by expressing the values of each attribute in a clear and concise manner. Its 2D visualization provides a comprehensive view of the data, making it easier to analyze and understand its various dimensions (Peng, 2022).

The method of radar chart for Multidimensional Data:

X = {X_1_, X_2_X_j_, ⋯ X_n_} is a multi-dimensional data set, and X_i_ {x_i1_, x_i2_, x_i3_x_iN_} is a N-dimensional vector. Use the radar chart when N⩾3 (Liu et al., 2008).

A method for evaluating the accessibility of a facility location using the area of a radar chart was provided by Takenaka and collaborators (Takenaka et al., 2018). The authors argue that the area of a radar chart is a more stable measure of accessibility than other measures.

The Area of the Radar (A_n_) was calculated with the Equation (2) where X_i_ = S_i_ {S1(%), S2(%), S3(%), S4(%), S5(%), S6(%)}.


(2) $$A_{n}\;\equiv \;\frac{1}{2}\;sin\;\frac{2\pi }{n}\;\sum_{i=1}^{n}x_{i-1}*x_{i\;}\;\;\;\;\left({\%}^{2}\right)\;\;\;\;\;\;\;$$

### 2.2 Instrumented chamber

The instrumented chamber was specially designed to collect gaseous samples while soybeans were growing. The chamber was equipped with sensors to measure the temperature (T in °C), relative humidity (RH in %), and CO_2_ concentration (CO_2_ in ppm). A computer fan was also installed inside the chamber to simulate wind (flux wind = 1 cubic feet per minute or 28.32 l/min). This chamber was also designed to administer irrigation without compromising insulation, with a valve connected directly to the ground. The pot containing the soil was covered with aluminum foil to avoid gas exchange between this medium and the chamber.

The chamber is positioned in an open and isolated area with solar illumination, and it is externally and internally instrumented for monitoring.

The technique for obtaining the gas was headspace.

Chamber indoor humidity was controlled with dry air (99% purity).

The monitoring of Temperature (°C), R.H. (%), and CO_2_ (ppm), both parameters measuring inside and outside of the chamber, was performed. The sensors used were an internal Vaisala CO_2_ Probe GMP252 sensor for measuring CO_2_ levels and an external Vaisala CO_2_ Probe GMP343 model, both operating in the range of 0 – 2,000 (ppm), an internal and an external digital thermometer with a resolution of 0.1°C, an indoor and an outdoor relative humidity sensor with a resolution of 0.5 (%). This experiment’s luminosity was natural and measured through a lux meter ranging from 0.001 (lumen/m²) to 19.9 K (lumen/m²).

Each internal sensor’s data in the chamber was obtained every five minutes and fed to a database. Acquisition software was developed, allowing the storage and reading of sensors in real time.

The temperature to experiment was ambient (monitored internally in the chamber and externally). Irrigation control occurred by calculating the desired amount of water, based on the volumetric moisture value of the wilting soybean point, concerning the soil used and the absence of water during the plant’s vegetative growth.

**Figure 4:**
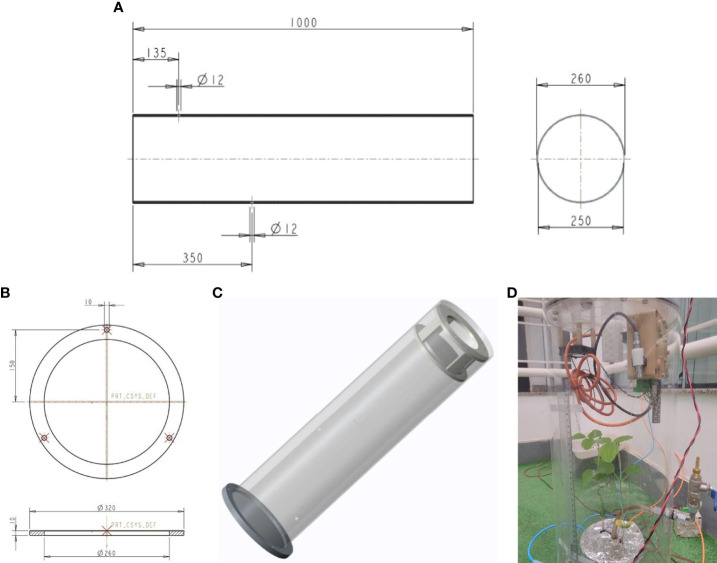
The illustration of dimension and configuration of the instrumented chamber developed and used in the experiment, **(A)** Diameter ext (d_ext_) = 260 (mm), Diameter int (d_int_) = 250 (mm), Thickness (Th) = 10 (mm), y = 570 (mm), Total volume = 27.93 (l); **(B)** details of the base to the chamber; **(C)** the draw of chamber assembled and **(D)** details of chamber that was developed with two plants of soybean growing inside. In this picture is possible to see the sensors, the fan installed, as well the inlet and outlet of the carrier gas and valve to control the water.

**Figure 4** illustrates the developed chamber used to allocate the plant and extract the emanating gas to be monitored in the experiment. The homemade chamber was built with a Poly(methyl methacrylate) (PMMA) tube, also known as acrylic or Plexiglas, with the Transmittance (DIN 5036, Part 3): ca. 92% (<0.05 (%) absorption in the visible range), Refractive index (ISO 489): 1.491, Max. permanent service temperature: 70 (°C), Material density (ISO 1183): 1.19 (g cm^-3^), Permeability coefficient (P0) @ 25 (°C) of oxygen 5.8 – 6.7 [(cm^3^ *·mm)/(m^2^ *·d·* atm)] and water vapor 1.7 [(cm^3^ *·mm)/(m^2^ *·d·* atm)] (Keller and Kouzes, 2017).

The baseline was obtained with an empty chamber before starting the soybean experiment. The Temp. (°C), R.H. (%), and CO_2_ (ppm) values were measured during 03 days before including the plant. Three measurements were performed with the E-Nose for the volume of gas samples of 500 (µl). Measurements were always performed at the same time as the experiment. The temperature inside the chamber fluctuated by 4.0°C, ranging from 23.0°C to 27.0°C. The relative humidity inside the chamber displayed a variation of 9.0%, ranging between 16% and 25%. Additionally, the concentration of CO_2_ inside the chamber showed a variation of 20.0 ppm_v_, ranging from 250 ppm_v_ to 270 ppm_v_.

### 2.3 Plant used in the experiment: soybean (*Glycine Max*)

The Brazilian soybean cultivar (*Glycine max L.* Merrill) BR-16 was used, treated, and subjected to drought under controlled conditions. The BR-16 soybean plants were irrigated during the growth phase and then subjected to drought for nine consecutive days.

The experiments were carried out during plant growth until the V5 stage of their vegetative cycle. Plants in V5 are approximately 25 to 30 (cm) tall and have six nodes, in which the leaves have unfolded leaflets.

Soybean specimens were studied in a laboratory environment, under controlled conditions, and with irrigation to verify water stress. The soil moisture for soybean emergence was between 15% and 20%, and the pot, with the plant, was included in the experiment chamber as was prepared.

The experiment was carried out with the soybean for 21 days (Days After Sowing (DAS) 11 – 32). Following the steps: DAS Irrigate: 11 – 20; DAS Not Irrigated: 21 – 32. The gas samples were obtained in the daylight hours [morning (09:00 – 10:00 a.m.) and afternoon (03:30 – 04:30 p.m.)].

### 2.4 Irrigation procedure

Irrigation was performed with Milli-Q deionized water (~12 MΩ*cm), through the adapted valve of the chamber.

The volume used to feed the plant was 100 (cm^3^) of Milli-Q water every two days.

The irrigation described was maintained for ten days, after which irrigation was completely stopped.

### 2.5 Soil

The soil used for this experiment was a dystrophic Red-Yellow Latosol (LVAd) with the following granulometry: Clay: 369 g/kg; Silt: 54 g/kg; Sand: 577 g/kg, with humidity at field capacity (considered at water tension of 10 KPa) of 0.295 cm^3^/cm^3^ and humidity at permanent wilting point (considered at water tension of -1,500 kPa) of 0.134 cm^3^/cm^3^.

The soil sample was obtained from Embrapa National Laboratory for Precision Agriculture (LANAPRE) at geographic coordinates 21°57’14” S and 47°51’08” W, 860 (m).

A study carried out by Ferreira et al. (2015) would have tested the covering of vessels with varied materials considering soybean and demonstrated that the isolation is effective in causing water losses to be the result only of transpiration, which was crucial to this experiment. Therefore, the soil was isolated with aluminum foil to reduce gas exchange between the medium of interest and the rhizosphere, and irrigation was administered directly into the ground. The volume of the constructed pot is V_pot_ = 8,100 (cm^3^). The dimensions of the pot used is the height (h_pot_)= 24.5 (cm), diameter (D_pot_)= 14.5 (cm), and the empty pot weight 682.9 (g).

The pot with the soil and the soybean, to conduct the experiment, was prepared with the following characteristics: dry soil weight (p_ds_) = 4,758 (g), the dry soil volume (V_ds_) = 8,090 (cm^3^) and the soil density (ρ_ds_) = 0.59 (g/cm^3^).

### 2.6 Data mining

The Weka™ (The University of Waikato Webpage, 2021; WEKA WIKI, 2021) tool was used for this work, and it was possible to apply several classification algorithms. K nearest neighbor (KNN) and the decision tree were used to evaluate the results from the database. A total of 500 gas samples from 12 soybeans were used to obtain the measured values with the E-Nose. The data was used to feed Weka database.

Data Mining is a subfield of machine learning that focuses on seeking patterns and behaviors within a database (Feyyad, 1996).

Several classic data mining strategies were considered and tested on the obtained database—for example, association algorithms, k-means clustering, k-nearest neighbors, logistic regression, and decision trees. The latter strategies returned more efficient and consistent results.

Decision trees represent a classification strategy based on a tree’s construction, where each node represents a logical test that separates a sample into different classes through parallel cuts in hyperplane space (Han et al., 2011; Loh, 2011).

After obtaining a well-structured and efficient tree, classifying a sample is a relatively simple task. This is one of the significant advantages of using this method when good results are achieved (Han et al., 2011; Wang et al., 2023).

## 3 Results

**Figure 5:**
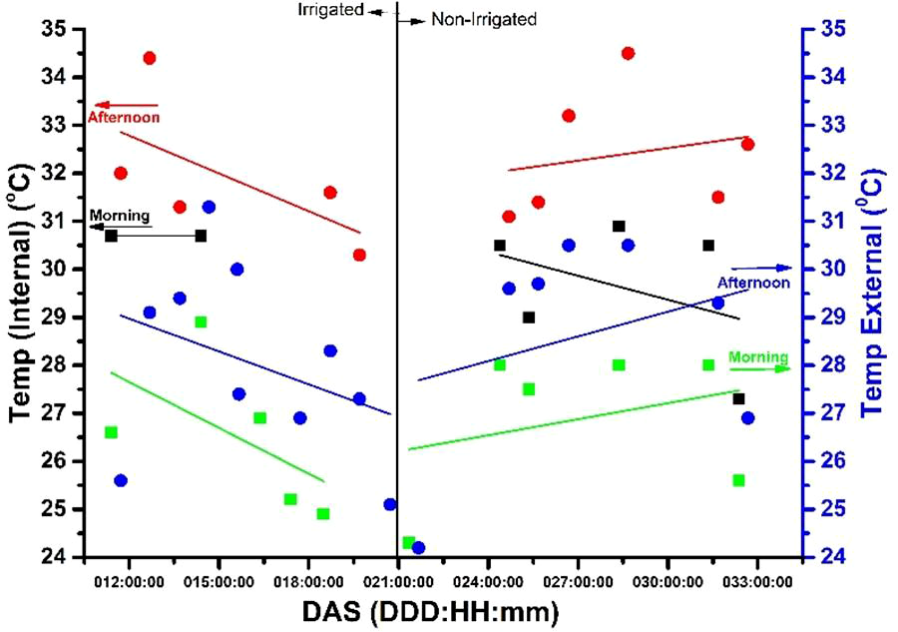
The temperature measurements, both external (blue y-axis) and internal (black y-axis), in degrees Celsius for both irrigated and non-irrigated conditions, in the morning (9:30 a.m.) and afternoon (3:30 p.m.).

**Figure 6:**
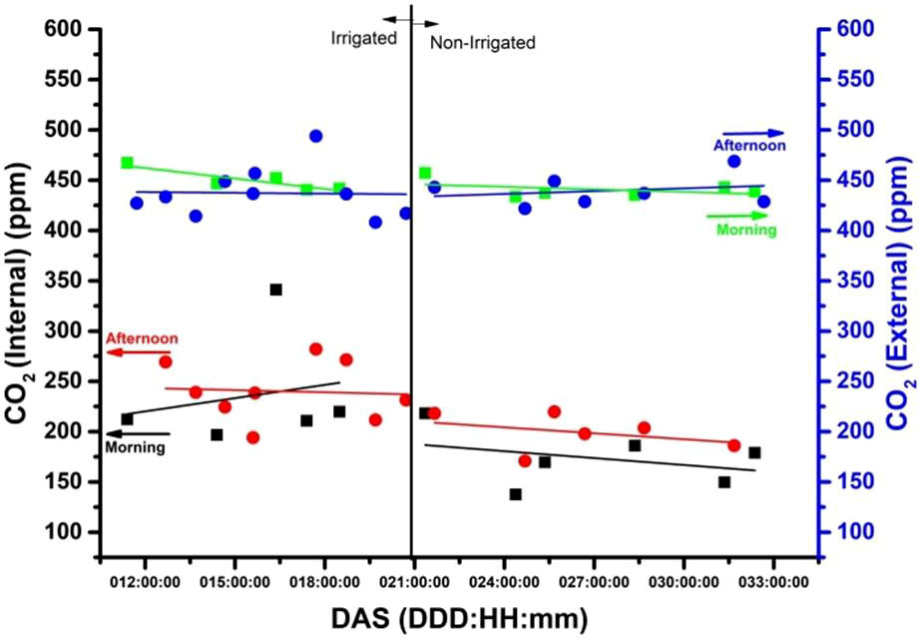
The external CO_2_ concentrations (ppm) measurement on the blue y-axis and the internal measurement (irrigated and non-irrigated) on the black y-axis, during the morning (9:30 am) and afternoon (3:30 pm).

**Figure 7:**
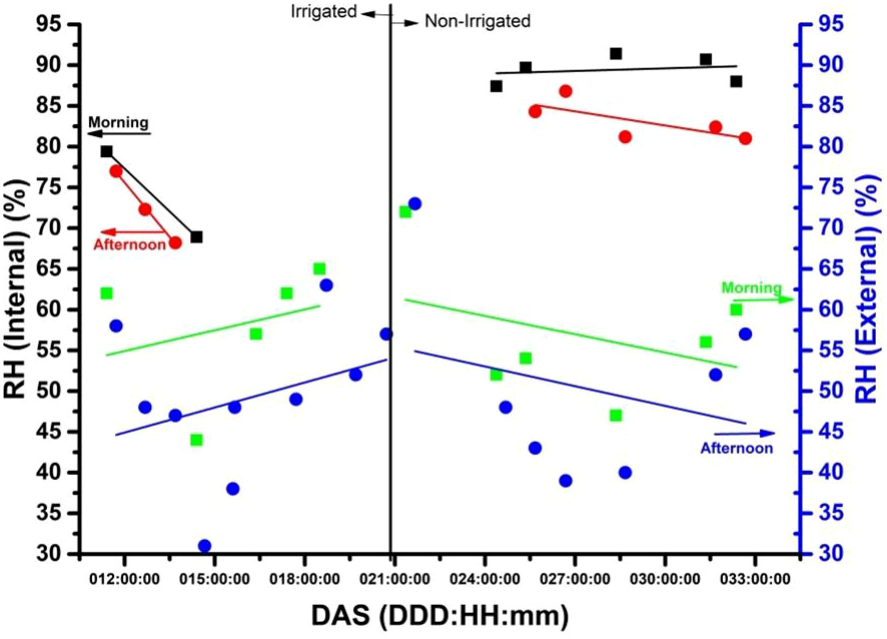
The internal relative humidity [R.H. (%)] in a plant chamber with and without irrigation for 31 days (about one month) after emergence (DAS), while also showing the external R.H. (%) in the laboratory during the morning (9:30 a.m.) and afternoon (3:30 p.m.).

**E**xperiment was conducted over a period of three years (from November 2017 to March 2020) and involved different soybeans subjected to water stress. This paper considers the results obtained from 22 days or roughly three weeks of experimentation. Specifically, the experiment was conducted between days after sowing (DAS) 10 to 32 for the plant. Sensors were placed both inside and outside the chamber to record the temperature (in °C), CO_2_ (ppm), R.H. (%), and LUX (external values only) during both morning and afternoon periods. **Figures 4**–**7** depict these results.

The x-axis on each figure shows the number of DAS from 11 to 32 DAS, while the y-axis displays the internal temperature in the chamber during both 9:30 a.m. (morning) and 3:30 p.m. (afternoon). The correlation between the x and y axis is represented by a trend line visible in red (afternoon) and black (morning), with a full red circle (afternoon) and a black square (morning) denoting the relationship. The graphs (3-6) depict two distinct segments of the experiment, with irrigation spanning from the 11^th^ to the 20^th^ day and no irrigation from the 21^st^ to the 32^nd^. On the right-hand side of the y-axis, the temperature of the laboratory environment in which the plant chamber is situated is illustrated. The deep blue full circle depicts the external temperature correlation in the afternoon, while the light green inverted square represents it in the morning. Throughout the 31-day experiment period after sowing (DAS), gas samples were collected at 9:30 a.m. and 3:30 p.m. for the E-Nose (**Figure 7**).

The relationship between temperature, relative humidity, CO_2_ concentration, and soybean growth in a closed chamber while experiencing water stress is complex and multifaceted (Smith et al., 2010). In a closed chamber with water stress, achieving optimal soybean growth requires a delicate balance between temperature, relative humidity, and CO_2_ concentration.

### 3.1 Internal and external temperature (°C) of chamber versus days after sowing of soybean

Temperature is perhaps the most crucial variable influencing the soybean’s metabolic rate and energy allocation. High temperatures can cause heat stress, reducing photosynthesis rates, impaired carbon fixation, and decreased yield (Yang et al., 2023). On the other hand, low temperatures can slow the soybean’s growth rate and delay its development. **Figure 5** shows us that there is clearly an increase in temperature, when compared to the temperature in the laboratory environment and the temperature inside the camera, with the plant inside, in irrigated and non-irrigated conditions. It is observed that the temperature is higher in the afternoon than in the morning.

**Figure 5**, presents temperature (°C) readings taken at 9:30 a.m. and 3:30 p.m. throughout the experiment, highlighting the temperature variance between the chamber and the experimental environment.

### 3.2 Internal and external CO_2_ levels (ppm) of chamber versus days after sowing of soybean

CO_2_ concentration (ppm) is a crucial variable for soybean growth as it affects photosynthesis. High CO_2_ concentrations (ppm) can enhance the soybean’s growth rate and yield, while low concentrations can reduce photosynthesis and growth. In **Figure 6**, the CO_2_ levels (ppm) inside and outside the chamber were measured using an internal and an external sensor respectively, providing insights into the experimental environment. **Figure 6** shows the level of CO_2_ concentration with the plant being irrigated and not irrigated. It is observed that the CO_2_ level, internal to the chamber, with the plant inside, through the trend line is below (~ 200 ppm) the CO2 concentration in the laboratory. It is verified for the non-irrigated period of the plant that there is a decline during the measurements carried out in the afternoons and mornings while the external CO_2_ remains practically constant.

### 3.3 Internal and external relative humidity [RH (%)] of chamber versus days after sowing of soybean

Relative humidity is also critical in soybean growth, affecting the plant’s water balance and transpiration rates. High humidity can increase the risk of disease and fungal infections, while low humidity can cause water stress and reduce the soybean’s growth rate.

To examine the impact of irrigation on the internal relative humidity [R.H. (%)] within a plant chamber, data was collected over a 31-day period following emergence (DAS). Two sets of data were analyzed, one with irrigation and one without. The resulting information is presented in **Figure 7** alongside the external R.H. (%) recorded within the laboratory. The graph in **Figure 7** shows the state of relative humidity [RH (%)] external and internal to the chamber. The behavior of RH (%), depending on the irrigated and non-irrigated plant stage, can be seen. Relative humidity is lower in the laboratory environment, while internally, there is a more significant variation for this stage, which was evident than in the non-irrigated condition. In this case, there is an increase in relative humidity, particularly in the morning, while in the afternoon, along the trend line (red), there is a brief decrease.

### 3.4 External luminosity [LUX (lumen/m²)] versus days after planting of soybean

**Figure 8:**
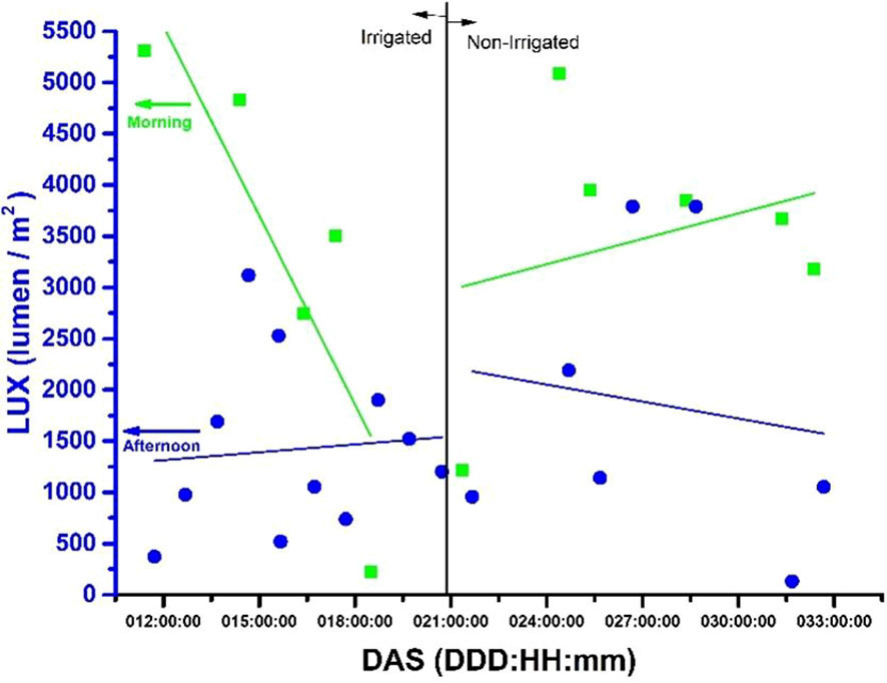
The graph shows the environmental luminosity measured in LUX (lumen/m2) for both irrigated and non-irrigated areas, during the morning (9:30 am) and afternoon (3:30 pm).

On **Figure 8**, is shows the relationship between LUX (lumen/m²) and days after planting (DAP) for plots that were irrigated and those that were not. The x-axis represents the number of DAP from 11 to 32, while the y-axis represents the LUX (lumen/m²). The correlation between the two axes is represented by a blue circle (afternoon) and a green square (morning), both accompanied by a trend line in blue (afternoon) and green (morning). LUX (lumen/m²) values were obtained at 9:30 in the morning and 3:30 in the afternoon. The figure is divided into two parts, with irrigation taking place between days 11 and 20 and no irrigation from days 21 to 32.

### 3.5 Radar area (%²) measure with electronic nose versus days after sowing of soybean

**Figure 9:**
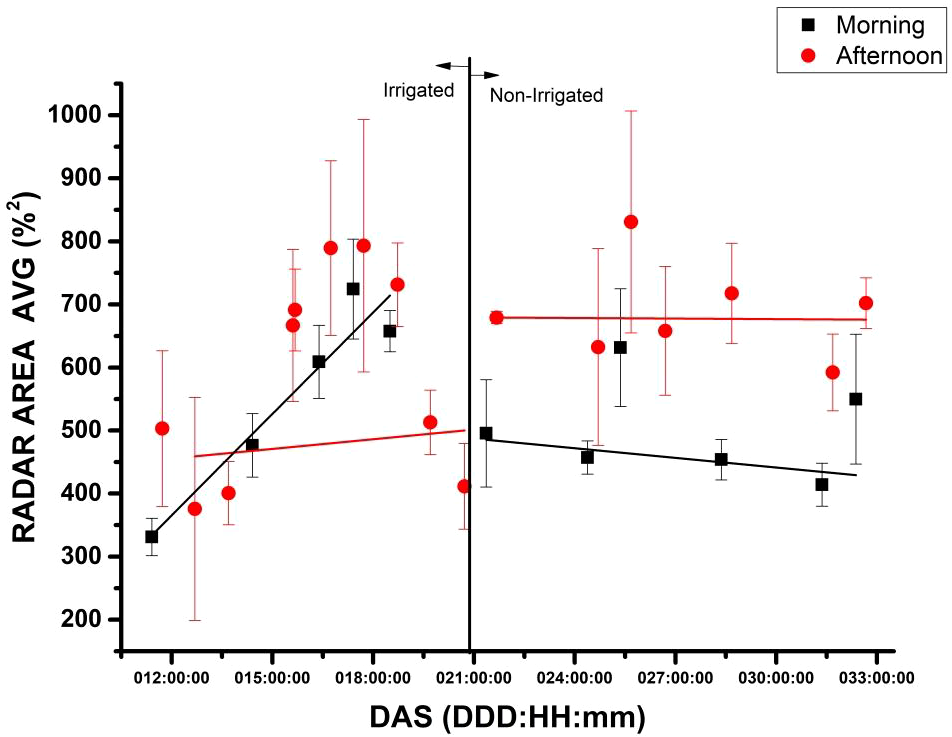
E-nose measurements of gas samples taken from a chamber containing soybeans during the DAS, using the average of radar area and standard deviation (n=3). The measurements are presented based on the time of day, either in the morning (9:30 a.m.) denoted by red circles or in the afternoon (3:30 p.m.) denoted by black squares. Moreover, the measurements are obtained from both irrigated and non-irrigated plants. For each DAS, gas samples are measured three times in both periods, i.e., the morning and afternoon to obtain the area radar measurement.

During the 31-day experiment period following the Day After Sowing (DAS), the Radar Area (U.A.) from the Electronic Nose (E-Nose) was recorded and is displayed in **Figure 9**. The x-axis of the graph shows the number of DAS from 11 to 32. At the same time, the y-axis displays the Area Radar (U.A.), based on the value of the intensity of electrical resistance (ohms), measured by six sensors, with the gas sample extracted from the chamber during both morning (9:30 a.m.) and afternoon (3:30 p.m.). The correlation between the x and y axis is demonstrated with a full red circle (afternoon) and a black square (morning), with the trend line visible in red (afternoon) and black (morning). **Figure 9** is segmented into two parts, with irrigation occurring between the 11th and 20th days and no irrigation from the 21st to the 32nd.

**Figure 10:**
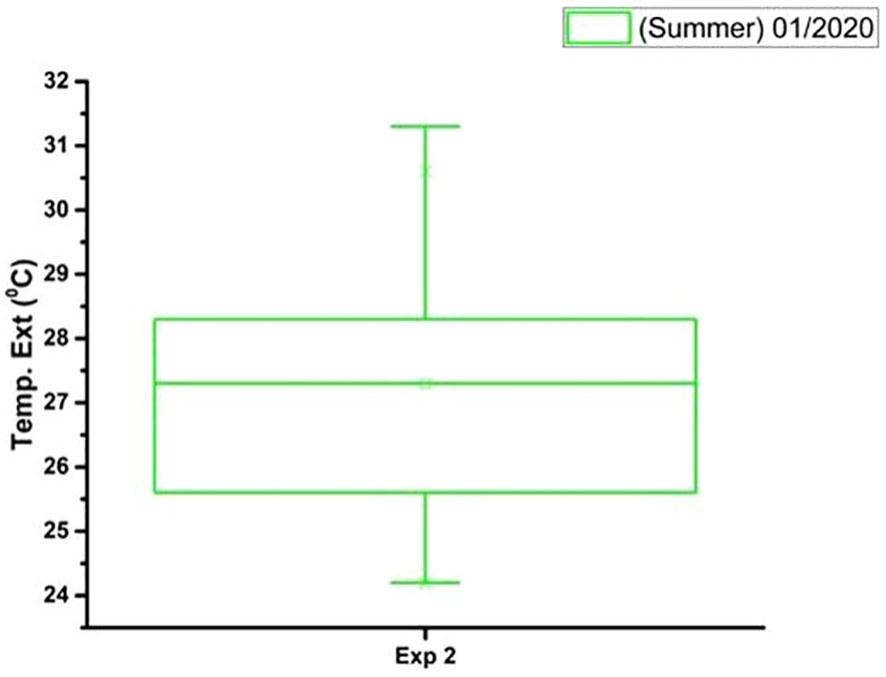
The box plot analysis of the external temperature (^0^C) during the period (Summer 01/2020) and total time of the experiment.

**Figure 11:**
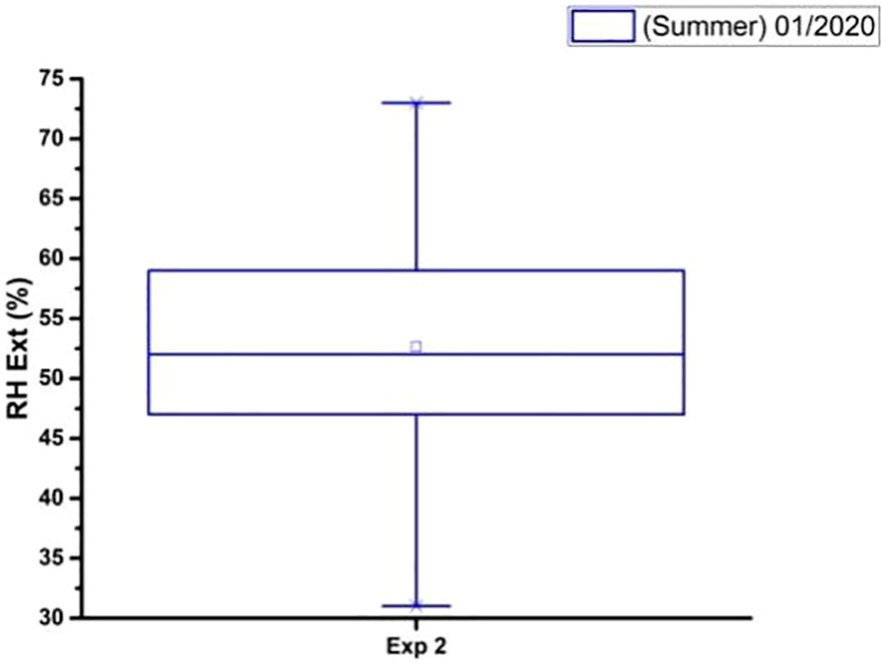
The box plot analysis of the external relative humidity (%) during the period (Summer 01/2020) and total time of the experiment.

**Figure 12:**
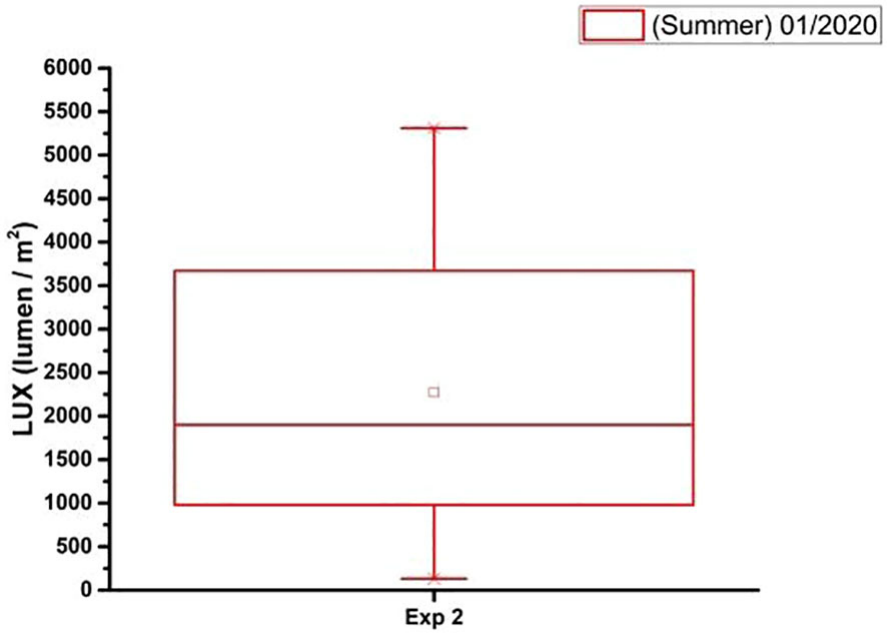
The box plot analysis of the laboratory luminosity (lumen/m^2^) obtained during the period (Summer 01/2020) and total time of the experiment.

In **Figures 10**–**12** there are graphs obtained from the local climatological station (São Carlos (SP), BRAZIL) showing the box plot of the parameter’s temperature (°C), relative humidity (%) and luminosity respectively, for the period (Summer 2020), where the experiments were carried out.

**Figure 13:**
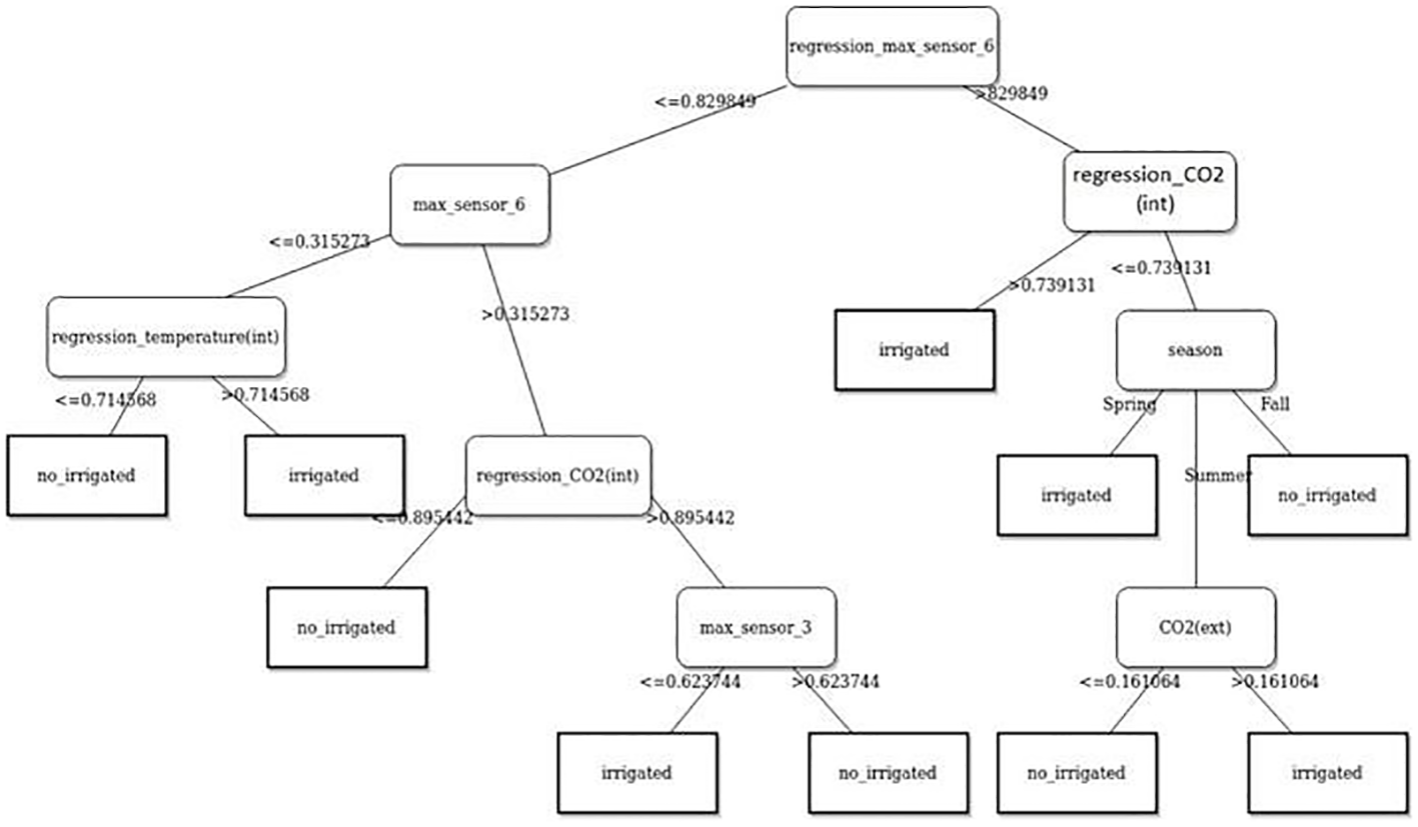
Decision tree learning extracted from test to plant 5.

The decision tree (DT) from the data mining and machine learning (ML) was used to visualize and explicate represent decision and decision making to the gas emanate from the plant in the state irrigated and not irrigated. **Figure 13** is showing the model to DT.

A series of experiments on twelve soybean specimens was performed and derived a highly efficient detection device that can accurately identify irrigation malfunctions in a staggering 94.4(%) of cases, as confirmed by a separate efficiency test database. The device solely relies on the data obtained from E-Nose readings, which are acquired by sampling the gas concentrated by soybeans.

**Table 2** shows the best learning results from sample classes (rows) and machine classifications (columns).

**Table 2:** Result of the best machine obtained.

	Decision Tree	
	Irrigated	Non irrigated
**Irrigated**	92.7%	7.3%
**Non irrigated**	5.7%	94.4%

## 4 Discussion

Reviewer 2: 2-3 key points for detailed discussion. Discuss the main findings rather than simply list the literature. The discussion part mainly focuses on the experimental results and the verification with mechanism analysis. Foi feito!

### 4.1 Instrumented chamber and analysis of the data obtained

The displayed temperature data in **Figure 5** exhibits the temperature in both the plant inside of the chamber and laboratory environment. This figure is separated into two sections, one showcasing irrigation between the 11^th^ to 20^th^ day and another without irrigation from the 21^st^ to the 32^nd^ day.

Throughout the observation period, the temperature in the chamber is consistently higher than that of the laboratory environment. The temperature disparity is more noticeable in the afternoon than in the morning. The chamber’s temperature reaches its highest point on the 11^th^ day and its lowest on the 32^nd^ day. Conversely, the laboratory environment’s temperature peaks on the 15^th^ day and hits its nadir on the 25^th^ day.

It is worth noting that irrigation has a potentially minor cooling impact on the chamber’s temperature by adding moisture to its surroundings.

According to the graph provided, it can be observed that the temperature within the chamber is controlled and differs from the temperature in the laboratory surroundings. This variation is primarily caused by water stress which leads to the closure of stomata, ultimately resulting in an increase in the leaf temperature. Mano et al. (2023) found in their works that water deficit led to reductions in stomata size and density in both maize and soybean leaves. These findings collectively support the idea that water stress-induced stomata closure contributes to an increase in soybean leaf temperature.

Based on the findings presented in **Figure 6**, it appears that the enclosed chamber experienced lower CO_2_ concentrations compared to the surrounding environment. This is likely due to the active photosynthesis process of the plants, which absorb carbon dioxide from the air. During the afternoon, when the plants were more actively engaged in photosynthesis due to intense sunlight, CO_2_ concentrations within the chamber were higher. However, after irrigation was stopped, the CO_2_ concentrations in the chamber decreased, which may have impacted the plant’s ability to photosynthesize effectively. It’s worth noting that CO_2_ concentrations outside the chamber remained stable throughout the day, possibly due to the experiment being carried out in laboratory conditions, where environmental factors that could affect measurements are strongly reduced.

The experiment clearly shows a difference in the amount of CO_2_ concentrated between the enclosed chamber and the external environment. This discrepancy is likely due to the plants in the chamber actively undergoing photosynthesis. When irrigation stopped, CO_2_ concentrations decreased because the plants were water-stressed and couldn’t photosynthesize as efficiently.

The influence of plant respiration on CO_2_ concentrations is distinct. While CO_2_ levels in the lab environment remained constant at around 450 ppm throughout the day, there was a noticeable change when comparing irrigated and non-irrigated plant conditions. The trend lines for CO_2_ concentrations demonstrate a variation between morning and afternoon during the irrigated phase.

In the absence of irrigation, there is a noticeable decline in CO_2_ levels during both morning and afternoon periods, indicating a higher release of carbon dioxide in the afternoon. However, the difference between the two situations is around 50 ppm. The external sensor, which serves as a reference, shows the impact of water stress on the plant’s behavior. Low CO_2_ concentrations suggest that the plants are actively absorbing CO_2_ from the surrounding environment rather than releasing it (Farquhar et al., 2001).

Higher CO_2_ concentrations reduce stomata opening, resulting in decreased transpiration. This is because plants can photosynthesize more effectively in an elevated CO_2_ environment, reducing the need for stomata opening to obtain the required CO_2_. Pallas in his investigation observed that increasing carbon dioxide content caused stomata closure and reduced transpiration rate in various plant species, including soybean (Pallas, 1965).

According to the findings illustrated in **Figure 7**, it was noted that the relative humidity [R.H. (%)] inside the chamber was greater than the R.H. (%) outside, especially in the morning. Gomes et al. (1987) discovered that elevated R.H. (%) leads to increased stoma opening in *Theobroma cacao* seedlings, implying that the internal R.H. (%) in the chamber may have surpassed the external R.H. (%). In the same way Arve and Torre also support this, showing that high R.H. promotes stomatal opening in tomato leaves (Arve and Torre, 2015). This can be attributed to the plant’s transpiration process, which introduced moisture to the air inside the chamber. Additionally, the R.H. (%) inside was higher during the irrigation phase compared to the no-irrigation period, as watering the soil and plants raised the amount of moisture in the air.

After analyzing the data presented in **Figure 8** pertaining to LUX (lumen/m^2^) values for 32 DAS, some noteworthy observations can be made. The highest LUX value recorded was 5,500 (lumen/m^2^), which was observed on DAS 11 in the afternoon under irrigated conditions. On the other hand, the lowest recorded LUX value was 1,000 (lumen/m^2^), which was observed on DAS 32 in the morning under non-irrigated conditions. Across all treatments, the average LUX value was 3,750 (lumen/m^2^). For irrigated conditions, the average LUX value was 4,000 (lumen/m^2^), whereas for non-irrigated conditions, it was 3,500 (lumen/m^2^). **Figure 7** shows the variability in terms of luminosity during the experiment period, related to the mornings and afternoons in which the measurements were taken.

According to the data from **Figure 9**, the Radar chart area (%^2^) values are higher in the afternoon compared to the morning. Daylight time can influence the gas emissions by soybean plants, particularly in terms of photosynthesis and respiration. During the irrigation period from 11 to 21 DAS, the Radar chart area (%^2^) values of irrigated and non-irrigated plants are similar. However, after the irrigation period ends on the 22nd day, the Radar chart area (%^2^) values for non-irrigated plants start to decrease. The increased values in the afternoon may be due to plant activity and transpiration during the day. Additionally, the slight increase in values over time may be due to plant growth and development. Similar values during the irrigation period could be attributed to adequate water supply in the soil for both groups of plants. The different behavior of plants in irrigated and non-irrigated conditions during the morning and afternoon periods, in non-irrigated conditions, the plants emit gasses as they grow. However, when the plants are under stress, the emission of gasses remains different in the morning and afternoon. The literature review suggests that as the plant grows from the vegetative stage (Vc) to the V5 stage, the number of leaves and stomatal density increases, leading to more significant gas exchanges. The study examines the non-irrigated portion and tracks environmental factors like temperature, humidity, internal and external CO_2_ levels, and luminosity. The findings indicate that the E-Nose detected a stage shift that aligns with previously cited research (Silva et al., 2020) that suggests a continual increase in stomatal conductance until soybeans reach the V3 or V4 stage.

### 4.2 Variation of radar area (%^2^) using the electronic-nose to monitor the whole plant under stress

When irrigation stops, non-irrigated plants may experience water stress which can result in a decrease in Radar chart area values.

This increase should continue if there are no interruptions in the water supply. It is important to note that the response to the absence of irrigation after the tenth day can vary depending on several physiological and environmental factors. The health and condition of the plant are crucial in this relationship, according to a study by Rodrigues and collaborators (Rodrigues et al., 2015).

All six sensors of the E-Nose detected the gas emitted by the plants, each with varying levels of sensitivity. Of these sensors, P10/1 (sensor 2) and P40/1 (sensor 4) showed the highest sensitivity (%). **Table 1** reveals that P10/1 is sensitive to combustible gasses, with hydrocarbons as the reference material, while P40/1 is sensitive to oxidizing gasses, with fluorine as the reference material. During irrigated conditions, from DAS 11 to 21, the peak sensitivity (%) to sensor P10/1 was -27.97 (%) ± 4.36 (%) and from non-irrigated conditions, from DAS 22 to 32, was -28.62 (%) ± 3.26 (%). Similarly, for sensor P40/1, during DAS from 11 to 21, the peak sensitivity (%) was -28.30 (%) ± 4.87 (%) and from non-irrigated conditions, during DAS 22 to 32, was -28.88 (%) ± 3.59 (%).

**Figure 9** displays the standard deviation of the values from the radar area for irrigated and non-irrigated soybean samples. Notably, the variance between morning and afternoon measurements is significant, with the largest standard deviation occurring in the afternoon time. The causes of this disparity could be attributed to various factors, including the plant’s physiological state, the environmental conditions during sample extraction, and the specific growth stage of the plant or errors in the syringe headspace. The most significant standard deviation occurred in the afternoon. On the 22^nd^ day of the experiment, during the afternoon measurements, there were weather conditions that included closed weather, rain, and rainy and cloudy conditions. The average luminosity (lumen/m^2^) measure during this time was 1,350 with a standard deviation of 1,050 (n=13), around 77% variation, much more than in the morning. In the morning the average luminosity (lumen/m^2^) measure was 3,461 with a standard deviation of 1,342 (n=11), around 39% variation. Soybeans are classified as a C3 plant, which means they use the Calvin cycle to photosynthesize. Abrupt variations in light intensity can stress soybean plants, especially affected by different light intensity treatments (Feng et al., 2019).

In tropical countries, afternoons, compared to mornings, tend to show a high temperature gradient, being significantly hotter. The photosynthetic rate of plants in general (which includes soybean plants) tends to decrease and gas exchange (respiration) increases in higher temperatures. The greatest variances observed in the afternoons (area radar graphs) are likely linked to greater exchange of gases.

The outcomes from the study suggest that the E-Nose has the potential to effectively monitor plant water stress.

The data on the climatic conditions of São Carlos (SP), BRAZIL to **Figures 10**–**12**, were provided by the conventional meteorological station of Embrapa Southeastern Livestock, located 21°57’42” S, 47°50’28” W, 860m.

**Figure 10** shows that the external temperature in January 2020 was relatively warm. The average temperature was above 25°C, and the standard deviation was relatively low. This means that the temperatures were generally consistent throughout the month. However, there were a few days with temperatures above 34°C in both experiments, and a few days with temperatures below 24°C.

From **Figure 11** the average relative humidity was 70% with a standard deviation of 5%. The median relative humidity was 70%, and the interquartile range (IQR) was 10%. This means that 50% of the relative humidity values were between 60% and 80%. The box plot analysis shows that the external relative humidity in January 2020 was relatively high.

With the analysis of **Figure 12** is possible to see that the external luminosity in January 2020 was relatively high. The average luminosity was 6,000 (lumen/m²) with a standard deviation of 500 (lumen/m²). The median luminosity was 5,500 (lumen/m²). This means that 50% of the luminosity values were between 4,500 (lumen/m²) and 7,500 (lumen/m²). The upper whisker extends to 8,500 (lumen/m²) and the lower whisker extends to 3,500 (lumen/m²). This means that there were a few days with luminosity values above 8,500 (lumen/m²) and a few days with luminosity values below 3,500 (lumen/m²).

The environmental conditions that were described by de data presented from the figures in **Figures 5**–**8**, **10**–**12**, in the DAS from 21 to 32, in which there was a lack of water and in its vegetative growth would have several impacts on the physiology of soybeans, particularly in terms of water stress. At the end of the experiment, the amount of moisture in the soil {measured as gravimetric soil moisture in percentage [θw (%)]} was determined. The sample of the dystrophic Red red-yellow latosol (LVAd) used in this investigation weighed 127.25 g. It was placed in an oven for 24 hours and regulated to a temperature of 102°C. After 24 hours, the dry weight of the soil was found to be 118.81 g, and the gravimetric soil moisture content was 7.1%.

Water stress is a significant factor affecting the physiology of soybeans. The absence of water for 10 days would likely cause significant stress to the soybean plants. According to the literature (Jumrani and Bhatia, 2019) it can lead to a decrease in photochemical quenching and electron transport rate, both of which are crucial for photosynthesis. According to a study, the photochemical quenching and electron transport rate in soybeans were significantly affected by temperature and water stress. The average photochemical quenching and electron transport rate values declined progressively as the growing temperatures increased.

The decrease in CO_2_ would also affect photosynthesis, as CO_2_ is a crucial component in the photosynthesis process. A lower concentration of CO_2_ can limit the rate of photosynthesis, potentially leading to reduced growth and yield.

The increase in relative humidity might help the soybean plants cope with the lack of water to some extent. Higher humidity can reduce the transpiration rate (water loss from plant leaves), which may help the plants conserve water. However, it’s also important to note that high humidity can create a conducive environment for certain plant diseases.

The decrease in ambient light intensity would likely impact photosynthesis as well. Light is another key component of photosynthesis and a decrease in light intensity can lead to a decrease in the rate of photosynthesis (Feng et al., 2019).

In response to these environmental conditions, soybeans would likely exhibit several physiological and biochemical adaptations. For instance, under water stress, the soluble sugar content in soybeans increases, presumably to reduce water-deficit-induced damage (Wang et al., 2022).

### 4.3 Machine learning technique, using decision tree, from evaluate the stress

The decision tree (DT) model shown in **Figure 13** is used to visualize decision making on gas emanate from plant in irrigated and not irrigated state. The DT is a hierarchical structure that starts with a root node and has branches that lead to child nodes. Each child node represents a decision point, and the branches leading away from the child node represent the possible outcomes of that decision. The DT terminates at leaf nodes, which represent the final decisions that can be made.

The DT in the image starts with the root node, which asks the question “Is regression_max_sensor 6<= 0.829849?” If the answer is yes, then the DT goes to the left child node, which asks the question “Is max_sensor 6<= 0.315273?” If the answer is yes, then the DT goes to the left child node, which predicts that the plant is irrigated. If the answer is no, then the DT goes to the right child node, which predicts that the plant is not irrigated.

If the answer to the root node question is no, then the DT goes to the right child node, which asks the question “Is regression_temperature(int)<= 0.714568?” If the answer is yes, then the DT goes to the left child node, which predicts that the plant is irrigated. If the answer is no, then the DT goes to the right child node, which predicts that the plant is not irrigated.

Using advanced technology such as the E-Nose and home built chamber, we were able to gather detailed data on plant irrigation methods. This data was carefully analyzed using Machine Learning algorithms, which ultimately resulted in a highly accurate detection rate of 94.4% for identifying inefficient irrigation practices in plants. This cutting-edge technology is revolutionizing the way we approach plant cultivation and ensuring that our crops are grown in the most efficient and sustainable ways possible.

## 5 Conclusion

After developing techniques (E-Nose and ML), methods, and data analysis evaluation, a distinctive water stress pattern was identified in soybean plants. The electronic nose signals, variations in mean and standard deviation, and machine learning proved highly effective in distinguishing plant physiology parameters in the whole plant, including: (i) - The growing plant; (ii) - Two scenarios: watered plants and water-stressed plants; (iii) - Gas collected from the chamber during DAS varied depending on the time of day (morning to 9:30 a.m. and afternoon to 3:30 p.m.); (iv) - The standard deviation of the radar area in each DAS, which suggested the influence of luminosity intensity due to soybean characteristics and variations in environmental conditions; (v) - The potential of using machine learning and decision trees to classify water stress status. These findings suggest that irrigation positively impacts the Area Radar (U.A.) values of the E-Nose. Therefore, it can be used as a non-invasive method to observe the impact of irrigation on whole plants.

Using machine learning and decision tree to detect the absence of irrigation with a 94.4% accuracy rate. The most common error identified was the misclassification of irrigated samples as non-irrigated. This type of error is considered less detrimental than overlooking a sample experiencing water stress. This allowed for the early identification of stress levels, which is a crucial factor in ensuring the healthy growth of plants. Furthermore, the preliminary outcomes acquired from the E-Nose signals and machine learning enabled the researchers to differentiate between the irrigated plant control and the water stress scenario and the impact of the two daylight periods.

The classifier has demonstrated stability when tested across various scenarios, even with different soybeans subjected to varying treatments used as the testing base. As a decision tree, it has the potential to integrate E-Nose and chamber data effectively to determine water stress. The practical development, implementation, and automation of these machines can be easily achieved.

Overall, these findings have significant implications for the field of plant science and could pave the way for more efficient and effective affordable techniques and methods of plant monitoring and care.

The effects observed in the two periods, mainly in the afternoon, really demand extensive research to reach an assertive conclusion. However, it is also understood that some insights (for future work) are useful and must be refined in the light of respiratory process and/or the photosynthetic rate.

Further studies should be carried out with controlled luminosity, aiming to investigate the effect of varying luminosity in a controlled manner, as well as carrying out studies on the use of E-Nose and machine learning with drought-tolerant wheat, one of the main diseases of wheat, caused by *Fusarium graminearum Schwabe*.

## Data availability statement

The raw data supporting the conclusions of this article will be made available by the authors, without undue reservation.

## Author contributions

PH: Writing – review & editing, Writing – original draft, Methodology, Investigation, Funding acquisition. MS: Writing – original draft, Formal analysis. EF: Writing – review & editing, Formal analysis. AT: Writing – original draft, Software, Formal analysis.

## References

[B1] Angelocci L. R. Marin F. R. Oliveira R. F. Righi E. Z. (2004). Transpiration, leaf diffusive conductance, and atmospheric water demand relationship in an irrigated acid lime orchard. Braz. J. Plant Physiol. Londrina 16, 53–64. doi: 10.1590/S1677-04202004000100008

[B2] Arve L. E. Torre S. (2015). Ethylene is involved in high air humidity promoted stomatal opening of tomato (Lycopersicon esculentum) leaves. Funct. Plant Biol. 42, 376–386. doi: 10.1071/FP14247 32480682

[B3] Basal O. Szabó A. (2020). Physiology, yield and quality of soybean as affected by drought stress. Asian J. Agric. Biol. 8, 247–252. doi: 10.35495/ajab.2019.11.505

[B4] Bray E. A. Bailey-Serres J. Weretilnyk E. (2000). “Responses to abiotic stresses,” in Biochemistry and Molecular Biology of Plants. Eds. Buchanan B. Gruissem W. Jones R. (Rockville, USA: American Society of Plant Physiologists), 1160.

[B5] Chatterjee S. Castro M. Feller J. F. (2013). An e-nose made of carbon nanotube-based quantum resistive sensors for the detection of eighteen polar/nonpolar VOC biomarkers of lung cancer. J. Mater. Chem. B 1, 4563–4575. doi: 10.1039/c3tb20819b 32261199

[B6] de Vries R. Muller M. van der Noort V. Theelen W. S. M. E. Schouten R. D. Hummelink K.. (2019). Prediction of response to anti-PD-1 therapy in patients with non-small cell lung cancer by electronic nose analysis of exhaled breath. Ann. Oncol. 30, 1660–1666. doi: 10.1093/annonc/mdz279 31529107

[B7] Embrapa Soja Soja em números (safra 2022/23). Available online at: https://www.embrapa.br/en/soja/cultivos/soja1/dados-economicos (Accessed June 2023).

[B8] Farquhar G. D. von Caemmerer S. Berry J. A. (2001). Models of photosynthesis. Plant Physiol. 125, 42–45. doi: 10.1104/pp.125.1.42 11154292 PMC1539321

[B9] Feng L. Raza M. A. Li Z. Chen Y. Khalid M. H. B. Du J.. (2019). The influence of light intensity and leaf movement on hotosynthesis characteristics and carbon balance of soybean. Front. Plant Sci. 9. doi: 10.3389/fpls.2018.01952 PMC6338029 30687355

[B10] Ferreira L.C. Neiverth W. Maronezzi L.F.F. Sibaldelli R.N.R. Nepomuceno A.L. Farias J.R.B.. (2015). Efficiency of cover materials in preventing evaporation in drought-stressed soybeans grown in pots. Rev. Cienc. Agrar. 58 (4), 359–356. doi: 10.4322/rca.1861

[B11] Feyyad U. M. (1996). Data mining and knowledge discovery: making sense out of data. IEEE Expert 11, 20–25. doi: 10.1109/64.539013

[B12] Fiorani F. Schurr U. (2013). Future scenarios for plant phenotyping. Annu. Rev. Plant Biol. 64, 267–291. doi: 10.1146/annurev-arplant-050312-120137 23451789

[B13] FOX Analyzer. (2000) Hardware User’s Guide – Manuel Number 001.

[B14] Garcia-Berrios E. Theriot J. C. Woodka M. D. Lewis N. S. (2013). Detection of ammonia, 2,4,6-trinitrotoluene, and common organic vapors using thin-film carbon black-metalloporphyrin composite chemiresistors. Sensors and Actuators B-Chemical 188, 761–767. doi: 10.1016/j.snb.2013.07.006

[B15] Gardner J. W. Bartlett P. N. (1994). A brief-history of electronic noses. Sensors Actuators B-Chemical 18, 211–220. doi: 10.1016/0925-4005(94)87085-3

[B16] Gomes A. R. S. Kozlowski T. T. Reich P. B. (1987). Some physiological responses of Theobroma cacao var. Catongo seedlings to air humidity. New Phytol. 107, 591–602. doi: 10.1111/j.1469-8137.1987.tb02929.x

[B17] Hale M. G. Orcutt D. M. (1987). The Physiology of Plants Under Stress Vol. i-xii (New Jersey, USA: ED: Wiley-Interscience, John Wiley & Sons, Inc), 1–206.

[B18] Han J. Kammber M. Pei J. (2011). Data Mining: Concepts and Techniques. 3. ed (Massachusetts: Morgan Kaufmann Publishers), 740.

[B19] Hazarika S. Choudhury R. Montazer B. Medhi S. Goswami M. P. Sarma U. (2020). “Detection of Citrus tristeza virus in mandarin orange using a custom-developed electronic nose system,” in IEEE Transactions on Instrumentation and Measurement, Vol. 6. 9010–9018. doi: 10.1109/TIM.2020.2997064

[B20] Jumrani K. Bhatia V. S. (2019). Interactive effect of temperature and water stress on physiological and biochemical processes in soybean. Physiol. Mol. Biol. Plants 25, 667–681. doi: 10.1007/s12298-019-00657-5 31168231 PMC6522612

[B21] Keller P. E. Kouzes R. (2017). Water Vapor Permeation in Plastics, Revision 1 Prepared for the U.S. Department of Energy under U.S. Department of Energy Contract DE-AC05-76RL01830 (USA: Pacific Northwesr National Laboratory).

[B22] Kiendler-Scharr A. Wildt J. Dal Maso M. Hohaus T. Kleist E. T. Mentel F.. (2009). New particle formation in forests inhibited by isoprene emissions. Nature 461, 381–384. doi: 10.1038/nature08292 19759617

[B23] Lambers H. Chapin F. S. Pons T. L. (2008). “Plant water relations,” in Plant Physiological Ecology (Springer, New York, NY). doi: 10.1007/978-0-387-78341-3_5

[B24] Liu W.–Y. Wang B.-W. Yu J.-X. Li F. Wang S.-X. Hong W.-X. (2008). “Visualization classification method of multi-dimensional data based on radar chart mapping,” in 2008 International Conference on Machine Learning and Cybernetics, Kunming, China: Proceedings of the Seventh International Conference on Machine Learning and Cybernetics. 857–862. doi: 10.1109/ICMLC.2008.4620524

[B25] Loh W.-Y. (2011). Classification and regression trees. WIREs Data Min. Knowl. Discovery 1, 14–23. doi: 10.1002/widm.8

[B26] Lozano J. Santos J. P. Horrillo M. C. (2005). Classification of white wine aromas with an electronic nose. Talanta 67, 610–616. doi: 10.1016/j.talanta.2005.03.015 18970214

[B27] Mano N. A. Madore B. Mickelbart M. V. (2023). Different leaf anatomical responses to water deficit in maize and soybean. Life 13, 290. doi: 10.3390/life13020290 36836647 PMC9966819

[B28] Manzoli A. Steffens C. Paschoalin R. T. Correa A. A. Alves W. F. Leite F. L.. (2011). Low-cost gas sensors produced by the graphite line-patterning technique applied to monitoring banana ripeness. Sensors (Basel) 11, 6425–6434. doi: 10.3390/s110606425 22163963 PMC3231435

[B29] Manzoli A. Steffens C. Paschoalin R. T. Graboski A. M. De Mello Brandão H. de Carvalho B. C.. (2019). Volatile compounds monitoring as indicative of female cattle fertile period using electronic nose. Sensors Actuators B: Chem. 282, 609–616. doi: 10.1016/j.snb.2018.11.109

[B30] Niederbacher B. Winkler J. B. Schnitzler J. P. (2015). Volatile organic compounds as non-invasive markers for plant phenotyping. J. Exp. Bot. 66, 5403–5416. doi: 10.1093/jxb/erv219 25969554

[B31] Pallas J. J.E. (1965). Transpiration and stomatal opening with changes in carbon dioxide content of the air. Science 147, 171–173. doi: 10.1126/science.147.3654.171 17790695

[B32] Pasqualotto G. Carraro V. Menardi R. Anfodillo T. (2019). Calibration of granier-type (TDP) sap flow probes by a high precision electronic potometer. Sensors 19, 2419. doi: 10.3390/s19102419 31137901 PMC6566514

[B33] Patakas A. Noitsakis B. Chouzouri A. (2005). Optimization of irrigation water use in grapevines using the relationship between transpiration and plant water status. Agriculture Ecosyst. Environ. 106, 253–259. doi: 10.1016/j.agee.2004.10.013

[B34] Peng W. (2022). Improved radar chart for lighting system scheme selection. Appl. Opt. 61, 5619–5625. doi: 10.1364/AO.455779 36255790

[B35] Persaud K. Dodd G. (1982). Analysis of discrimination mechanisms in the mammalian olfactory system using a model nose. Nature 299, 352–355. doi: 10.1038/299352a0 7110356

[B36] Ratzmann G. Zakharova L. Tietjen B. (2019). Optimal leaf water status regulation of plants in drylands. Sci. Rep. 9, 3768. doi: 10.1038/s41598-019-40448-2 30842586 PMC6403219

[B37] Rodrigues F. A. Fuganti-Pagliarini R. Marcolino-Gomes J. Nakayama T. J. Molinari Correa H. B. Lobo F. P.. (2015). Daytime soybean transcriptome fluctuations during water deficit stress. BMC Genomics 16, 505. doi: 10.1186/s12864-015-1731-x 26149272 PMC4491896

[B38] Schaller E. Bosset J. O. Escher F. (1998). Electronic noses and their application to food. Food Sci. Technology-Lebensmittel-Wissenschaft Technologie 31, 305–316. doi: 10.1006/fstl.1998.0376

[B39] Sharma C. Barkataki N. Sarma U. (2023). A deep neural network with electronic nose for water stress prediction in Khasi Mandarin Orange plants. Measurement Sci. Technol. 34. doi: 10.1088/1361-6501/acf8e3

[B40] Silva J. A. Santos P. A. B. Carvalho L. G. Moura E. G. Andrade F. R. (2020). Gas exchanges and growth of soybean cultivars submitted to water deficiency. Pesquisa Agropecuária Trop. 50, e58854. doi: 10.1590/1983-40632020v5058854

[B41] Sinclair T. R. Messina C. D. Beatty A. Samples M. (2010). Assessment across the United States of the benefits of altered soybean drought traits. Agron. J. 102, 475–482. doi: 10.2134/agronj2009.0195

[B42] Smith S. E. Facelli E. Pope S. Smith F. A. (2010). Plant performance in stressful environments: interpreting new and established knowledge of the roles of arbuscular mycorrhizas. Plant Soil 326, 3–20. doi: 10.1007/s11104-009-9981-5

[B43] Steffens C. Leite F. L. Manzoli A. Sandoval R. D. Fatibello O. Herrmann P. S. P. (2014). Microcantilever sensors coated with a sensitive polyaniline layer for detecting volatile organic compounds. J. Nanoscience Nanotechnology 14, 6718–6722. doi: 10.1166/jnn.2014.9348 25924322

[B44] Sun Y. Guo H. Yuan L. Wei J. Zhang W. Ge F. (2015). Plant stomatal closure improves aphid feeding under elevated CO_2_ Global Change Biology. Global Change Biology 21 (7), 2739–2748. doi: 10.1111/gcb.12858 25581722

[B45] Takenaka T. Nakamura K. Ukai T. Ohsawa Y. (2018). Stability of the area of radar chart to evaluate the accessibility of facility location. J. City Plann. Institute Japan 53 (3), 640–645. doi: 10.11361/journalcpij.53.640

[B46] Tan P. N. Kumar V. Srivastava J. (2006). “Selecting the right interestingness measure for association patterns,” in KDD '02: Proceedings of the eighth ACM SIGKDD international conference on Knowledge discovery and data mining. Edmonton: KDD - 2002 Proceedings of the Eight ACM SIGKDD International Conference on Knowledge Discovery and Data Mining, Association for Computing Machinery. 32–41.

[B47] THE UNIVERSITY OF WAIKATO WEBPAGE. Available online at: https://www.cs.waikato.ac.nz/ml/weka/ Accessed March, 2021).

[B48] Vernat-Rossi V. Garcia C. Talon R. Denoyer C. Berdague J. L. (1996). Rapid discrimination of meat products and bacterial strains using semiconductor gas sensors. Sens. And Actuat. B 37, 43–48. doi: 10.1016/S0925-4005(97)80070-6

[B49] Wang X. Wu Z. Zhou Q. Wang X. Song S. Dong S. (2022). Physiological response of soybean plants to water deficit. Front. Plant Sci. 12. doi: 10.3389/fpls.2021.809692 PMC8842198 35173752

[B50] Wang X. Zhou Y. Zhao Z. Feng X. Wang Z. Jiao M. (2023). Advanced algorithms for low dimensional metal oxides-based electronic nose application: A review. Crystals 13, 615. doi: 10.3390/cryst13040615

[B51] Wei W. Li J. Huang L. (2017). Discrimination of producing areas of Astragalus membranaceus using electronic nose and UHPLC-PDA combined with chemometrics. Czech J. Food Sci. 35, 40–47. doi: 10.17221/126/2016-CJFS

[B52] WEKA WIKI. Available online at: https://waikato.github.io/weka-wiki/ (Accessed March, 2021).

[B53] Wijewardana C. Alsajri F. A. Irby J. T. Krutz L. J. Golden B. Henry W. B.. (2019). Physiological assessment of water deficit in soybean using midday leaf water potential and spectral features. J. Plant Interact. 14, 533–543. doi: 10.1080/17429145.2019.1662499

[B54] Yang L. Song W. Xu C. Sapey E. Jiang D. Wu C. (2023). Effects of high night temperature on soybean yield and compositions. Front. Plant Sci. 17. doi: 10.3389/fpls.2023.1065604 PMC9987466 36890900

[B55] Zhao C. Liu B. Piao S. Wang X. Lobell D. B. Huang Y.. (2017). Temperature increase reduces global yields of major crops in four independent estimates. Proc. Natl. Acad. Sci. U.S.A. 114, 9326–9331. doi: 10.1073/pnas.1701762114 28811375 PMC5584412

